# Silencing of miR-34a Attenuates Cardiac Dysfunction in a Setting of Moderate, but Not Severe, Hypertrophic Cardiomyopathy

**DOI:** 10.1371/journal.pone.0090337

**Published:** 2014-02-27

**Authors:** Bianca C. Bernardo, Xiao-Ming Gao, Yow Keat Tham, Helen Kiriazis, Catherine E. Winbanks, Jenny Y. Y. Ooi, Esther J. H. Boey, Susanna Obad, Sakari Kauppinen, Paul Gregorevic, Xiao-Jun Du, Ruby C. Y. Lin, Julie R. McMullen

**Affiliations:** 1 Baker IDI Heart and Diabetes Institute, Melbourne, Victoria, Australia; 2 Santaris Pharma, Horsholm, Denmark; 3 Department of Haematology, Aalborg University Hospital, Copenhagen, Denmark; 4 Ramaciotti Centre for Genomics, School of Biotechnology & Biomolecular Sciences, University of New South Wales, Sydney, New South Wales, Australia; 5 Departments of Medicine Monash University, Clayton, Victoria, Australia; 6 Departments of Physiology, Monash University, Clayton, Victoria, Australia; Rutgers New Jersey Medical School, United States of America

## Abstract

Therapeutic inhibition of the miR-34 family (miR-34a,-b,-c), or miR-34a alone, have emerged as promising strategies for the treatment of cardiac pathology. However, before advancing these approaches further for potential entry into the clinic, a more comprehensive assessment of the therapeutic potential of inhibiting miR-34a is required for two key reasons. First, miR-34a has ∼40% fewer predicted targets than the miR-34 family. Hence, in cardiac stress settings in which inhibition of miR-34a provides adequate protection, this approach is likely to result in less potential off-target effects. Secondly, silencing of miR-34a alone may be insufficient in settings of established cardiac pathology. We recently demonstrated that inhibition of the miR-34 family, but not miR-34a alone, provided benefit in a chronic model of myocardial infarction. Inhibition of miR-34 also attenuated cardiac remodeling and improved heart function following pressure overload, however, silencing of miR-34a alone was not examined. The aim of this study was to assess whether inhibition of miR-34a could attenuate cardiac remodeling in a mouse model with pre-existing pathological hypertrophy. Mice were subjected to pressure overload via constriction of the transverse aorta for four weeks and echocardiography was performed to confirm left ventricular hypertrophy and systolic dysfunction. After four weeks of pressure overload (before treatment), two distinct groups of animals became apparent: (1) mice with moderate pathology (fractional shortening decreased ∼20%) and (2) mice with severe pathology (fractional shortening decreased ∼37%). Mice were administered locked nucleic acid (LNA)-antimiR-34a or LNA-control with an eight week follow-up. Inhibition of miR-34a in mice with moderate cardiac pathology attenuated atrial enlargement and maintained cardiac function, but had no significant effect on fetal gene expression or cardiac fibrosis. Inhibition of miR-34a in mice with severe pathology provided no therapeutic benefit. Thus, therapies that inhibit miR-34a alone may have limited potential in settings of established cardiac pathology.

## Introduction

Cardiovascular disease remains the leading cause of morbidity and mortality worldwide and whilst current drugs (e.g. angiotensin-converting enzyme inhibitors, beta blockers) improve symptoms and reduce hospital admissions, the prevalence of cardiovascular disease is still increasing, highlighting the need for the identification of novel and efficacious therapies that can prevent cardiovascular disease. microRNAs (miRNAs) are an abundant class of small, non-coding RNAs that target partially complementary sequences in the 3’ untranslated region of target mRNA, leading to mRNA cleavage and/or translational repression [1]. Whilst miRNAs regulate a wide range of biological processes, recent studies have unveiled critical roles of miRNAs in cardiovascular disease (reviewed in [2]–[6]). These studies highlight their potential as novel therapeutic targets. miRNA inhibitors or antimiRs have been shown to be efficient in silencing miRNAs in mice [7], and non-human primates [8], and the first miRNA-targeted drug, miravirsen, has advanced into clinical phase 2 trials for the treatment of hepatitis C virus infection [9]. Although miRNA based therapies for cardiovascular disease have not yet reached clinical trials, a number of successful preclinical studies in animal models of heart failure, cardiac hypertrophy and remodeling highlight the translational potential of targeting miRNAs in human heart failure [10]–[15]. Most preclinical studies have focused on inhibiting individual miRNAs in the heart [11], [13], [15]. However, more recent studies have demonstrated the therapeutic potential of targeting disease-implicated miRNA-families [10], [14], [16], [17]. Though a potential disadvantage of inhibiting an entire miRNA family is the increased risk of generating off-target effects. Thus it is important to assess whether the effects of silencing miRNA families can yield more therapeutic benefit in settings of cardiac stress than the targeting of individual miRNAs.

We and others have previously shown that expression of miR-34a is elevated in settings of cardiac stress [10], [18] and ageing [11], and that miR-34 family members, 34b and 34c, are also elevated in the mouse heart following a cardiac insult [10], [18]. Furthermore, expression of miR-34 family members was found to be elevated in cardiac tissue from patients with heart disease [19], [20]. We recently found that inhibition of the miR-34 family, but not miR-34a alone, displayed a therapeutic benefit in a chronic model of myocardial infarction (MI, with pre-existing cardiac dysfunction and significant left ventricular (LV) remodeling; antimiR delivered 2 days after MI) [10]. Inhibition of the miR-34 family also improved cardiac function and attenuated LV remodeling in a mouse model with pre-existing pathological cardiac remodeling and dysfunction due to pressure overload by transverse aortic constriction (TAC) [10], however the therapeutic impact of inhibiting miR-34a alone was not assessed in that study. More recently, it was shown that inhibition of miR-34a prevented cardiac contractile dysfunction, and reduced apoptosis and fibrosis following acute MI, but the first dose of the miRNA-based therapy was administered 3 hours after acute MI, before any chronic LV remodeling had occurred [11]. Collectively, these findings suggest that inhibition of miR-34a might be beneficial in acute settings of cardiac stress or conditions with moderate pathology, but not chronic or severe settings. Given the current enthusiasm and anticipation regarding therapeutic development of miR-34a and miR-34 family-targeted antimiRs [11], [21]–[23], and the differences in cardiac protection in acute versus chronic settings [10], [11], it is important to assess the therapeutic potential of inhibiting miR-34a in more sustained pathological settings. This is also important because a challenge facing miRNA-based therapies is the vast number of predicted targets which could result in off-target effects. Since the miR-34 family has approximately 31–55% more targets in humans (which is dependent on the particular target prediction algorithm used) than miR-34a alone, interventions that modulate the entire miRNA family have greater theoretical potential to generate off-target effects. Thus, if manipulation of a single miRNA can provide adequate cardiac protection, this approach may be an advisable alternative means of obtaining a therapeutic effect with lower risk of off-target complications.

In the current study, we assessed the therapeutic potential of inhibiting miR-34a in a mouse model with pre-existing pathological hypertrophy and systolic dysfunction due to pressure overload induced by TAC. We report here, that administration of a locked nucleic acid (LNA)-antimiR-34a in a model of pressure overload-induced hypertrophic cardiomyopathy with moderate systolic dysfunction was able to prevent further deterioration in cardiac function over an eight week period. In contrast, this approach was unable to attenuate pathological remodeling in a model of pressure overload with severe systolic dysfunction, whereby systolic function continued to decline eight weeks after treatment.

## Methods

### Experimental animals

All experiments using animals were conducted in accordance with the Australian code of practice for the care and use of animals for scientific purposes (National Health & Medical Research Council of Australia, 8^th^ Edition, 2013). Animal care and experimental procedures were approved by the Alfred Medical Research and Education Precinct’s Animal Ethics Committee. All surgery was performed under anesthesia (ketamine:xylazine:atropine) with post-operative analgesia (carprofen), and all efforts were made to minimize animals’ discomfort.

### Pressure overload

Adult (12 week old) male C57BL/6 mice were subjected to a pressure overload surgery induced TAC as previously described [24]. In brief, mice were anaesthetized with ketamine:xylazine:atropine (100:20:1.2 mg/kg, i.p.), administered an analgesic (carprofen, 5 mg/kg, s.c.) and intubated for ventilation. A sternectomy was performed to access the aorta and a non-absorbable 5–0 braided silk suture was tied around the aorta between the right innominate and left carotid arteries, causing a constriction of approximately 65% using a 0.45 mm probe as a guide. Mice were monitored and studied for 12 weeks. Controls were unoperated aged matched male mice.

### LV structure and function

Transthoracic echocardiography (M-mode two-dimensional echocardiography) was performed on anaesthetized mice (1.8% isoflurane, inhalation) using a Philips iE33 ultrasound machine with a 15 mHz liner array transducer, prior to surgery (baseline), four weeks post TAC, and endpoint echocardiography was performed eight weeks post treatment (i.e. 12 weeks post TAC). LV chamber dimensions (LV end-diastolic dimension, LVEDD; LV end-systolic dimension, LVESD), LV wall thicknesses (LV posterior wall, LVPW; interventricular septum, IVS), heart rate (HR), fractional shortening (FS, calculated as [(LVEDD – LVDSD)/LVEDD] × 100%) and ejection fraction (EF, calculated as [(LVEDD^3^ – LVDSD^3^)/LVEDD^3^] × 100%) were analyzed offline using dedicated software (ProSolv Cardiovascular Analyzer version 3.5; ProSolv, Indianapolis, IN) obtained from M-mode traces.

### Classification of cardiac phenotype

The TAC model is associated with pathological hypertrophy and systolic dysfunction within four weeks of surgery [25], [26]. Cardiac function was assessed before surgery and at four weeks post-surgery. At four weeks post surgery, the analysis of echocardiography parameters (i.e. FS, EF) was used to define mice as exhibiting either a moderate (n = 9) or severe phenotype (n = 6).

### LNA-oligonucleotide synthesis

The antimiR-34a was synthesized as a LNA:DNA mixmer with a complete phosphorothioate backbone (Santaris Pharma A/S, Denmark). The sequence of the 15-mer antimiR-34a was: 5′ – AgCtaAGacACTgCC – 3′ (LNA uppercase, DNA lowercase), and was specifically designed to target miR-34a, as previously reported [10]. The 15-mer LNA-control was synthesized (Santaris Pharma A/S, Denmark) with the following sequence: 5′ – TcAtcCTatAtGaCA – 3’ (LNA uppercase, DNA lowercase). The LNA-control sequence was randomly chosen using a well-validated and established antimiR design. It was checked against a number of databases and shown not to have any perfect match binding sites in the transcriptome. Validation with *in vitro* and *in vivo* assays demonstrated that the control did not differ from un-transfected/mock or saline samples [27].

### In vivo delivery of LNA-antimiR oligonucleotides

Four weeks post TAC (after echocardiography), TAC mice were randomized into control or treated groups. Mice were subcutaneously administered either a LNA-control or LNA-antimiR-34a (25 mg/kg/day) over three consecutive days and left for a period of eight weeks (Figure S1). Studies conducted in our laboratory have demonstrated that LNA-control itself has no effect on body weight, tibia length, and other organ weights (including heart, atria, lung, kidney and liver) compared with the same dosing regime of saline [comparison of LNA-control (3 daily doses: 25 mg/kg/day) or saline and 8 week follow-up in adult C57BL/6 mice; Bernardo BC., unpublished data].

Control mice were unoperated, untreated aged matched male mice. Our previous study demonstrated that administration of LNA-antimiR-34a had no effect in sham mice when compared to LNA-control mice, and that this dosing regimen is sufficient to silence miRNAs in the heart for at least two months [10]. In male C57BL/6 mice, administration of LNA-antimiR-34a had no effect on body weight (BW), tibial length (TL), heart weight (HW), atrial weight (AW) and lung weight (LW) after two months (Table S1). Therefore we used unoperated/untreated mice as controls.

### RNA and protein isolation

Total RNA was isolated from frozen mouse ventricles using TRI Reagent (Sigma-Aldrich, St Louis, MO) and quantitated on a Nanodrop Spectrometer (Thermo Scientific, Waltham, MA). For protein lysates, frozen mouse ventricles were homogenized in a lysis buffer (10% glycerol, 137 mM NaCl, 20 mM Tris-HCl (pH 7.4), 20 mM NaF, 10 mM EDTA, 1 mM EGTA, 1 mM sodium pyrophosphate, 1 mM vanadate, 1 mM PMSF, 4 µg/mL pepstatin, 4 µg/mL aprotinin, 4 µg/mL leupeptin) and total protein concentration was determined using a Pierce micro protein assay kit (Thermo Scientific).

### Quantitative PCR (qPCR)

For mRNA expression analysis, 2 µg of total RNA was reverse transcribed using the High Capacity RNA-to-cDNA kit (Life Technologies, Carlsbad, CA) according to manufacturer’s recommendations. qPCR was performed using TaqMan probes (Life Technologies) and amplified on an Applied Biosystems 7500 real-time PCR instrument according to manufacturer’s instructions. Hypoxanthine phosphoribosyltransferase 1 (*Hprt1)* was used to standardize for cDNA concentration and data was analyzed using the 2^-▵▵Ct^ method of quantification [28]. For miRNA expression analysis, 50 ng of total RNA was reversed transcribed for each miRNA of interest using TaqMan® MicroRNA Reverse Transcription Kit (Life Technologies) according to manufacturer’s recommendations. qPCR was performed using TaqMan® MicroRNA Assays (Life Technologies) according to manufacturer’s instructions. Expression was normalized against snoU6 and data analyzed using the 2^-▵▵Ct^ algorithm [28].

### Northern blotting

10 µg of total RNA was separated on a formaldehyde denaturing agarose gel and transferred to a Hybond-N membrane (GE Healthcare, Pittsburgh, PA) in 20X SSC by upward capillary transfer, and probed for atrial natriuretic peptide (*Anp*), B type natriuretic peptide (*Bnp*), β myosin heavy chain (*β-MHC*), sarcoplasmic/endoplasmic reticulum Ca^2+^-ATPase 2a *(Serca2a),* and glyceraldehyde-3-phosphate dehydrogenase *(Gapdh)* as previously described [29]. Membranes were stripped and re-probed for subsequent analyses. Signals were quantitated using ImageJ 1.44p pixel analysis (NIH Image Software) and data normalized to a control value of 1.

### Western blotting

20 µg of protein lysates were separated by SDS-PAGE using pre-cast 4–12% Bis-Tris gels (Life Technologies), blotted onto nitrocellulose membrane (BioRad, Hercules, CA), incubated with antibody overnight (VEGF-A, Santa Cruz, Santa Cruz, CA, sc-152, 1∶500; vinculin, Sigma Aldrich, clone hVIN-1, V9131, 1∶2000; GAPDH, Santa Cruz, sc-32233, 1∶5000) and detected by chemiluminescence. Membranes were stripped and re-probed for subsequent analyses. Signals were quantitated using ImageJ 1.44p pixel analysis (NIH Image Software) and data normalized to a control value of 1 [30].

### Fibrosis

Ventricle samples were fixed in 4% paraformaldehyde and paraffin embedded for histological analysis at 6 µm cross-sections. Cardiac collagen deposition/interstitial fibrosis was assessed by Masson’s trichrome stain (Alfred Pathology, Melbourne, Australia). Images of the LV were obtained using an Olympus light microscope at 40x magnification. Collagen stained blue, which was measured and analyzed using Olympus Image-Pro Plus version 6.0. The percentage fibrosis was calculated by dividing the total area of collagen by the total area of the LV and multiplying by 100%. Data were normalized to a control value of 1 and presented as a fold change.

### Cardiomyocyte area

Ventricle samples were fixed in 4% paraformaldehyde and paraffin embedded for cardiomyocyte area analysis at 4 µm cross-sections. Cell membranes were stained with Fluorescein Wheat Germ Agglutinin (WGA; Vector Laboratories Inc; Burlingame, CA, USA). Cardiomyocyte size was analysed using ImageJ software (Bethesda, MD, USA) by averaging the measurements (arbitrary units) of at least 60 individual cardiomyocytes per heart, as previously described [10].

### Statistical analyses

Statistical analyses was performed using StatView (Version 5.0.1, SAS Institute Inc.). Results are presented as mean ± SEM. Differences between groups were identified using one-way analysis of variance (ANOVA) followed by *Fisher’s* post-hoc tests, unless otherwise indicated. Unpaired t-tests were used when comparing two groups for a single measure. A value of P<0.05 was considered significant. All relative units are expressed as a fold change with the relevant control group normalized to 1.

## Results

### Distinct remodeling and function in a moderate and severe model of pressure overload induced hypertrophy

**Figure 1:**
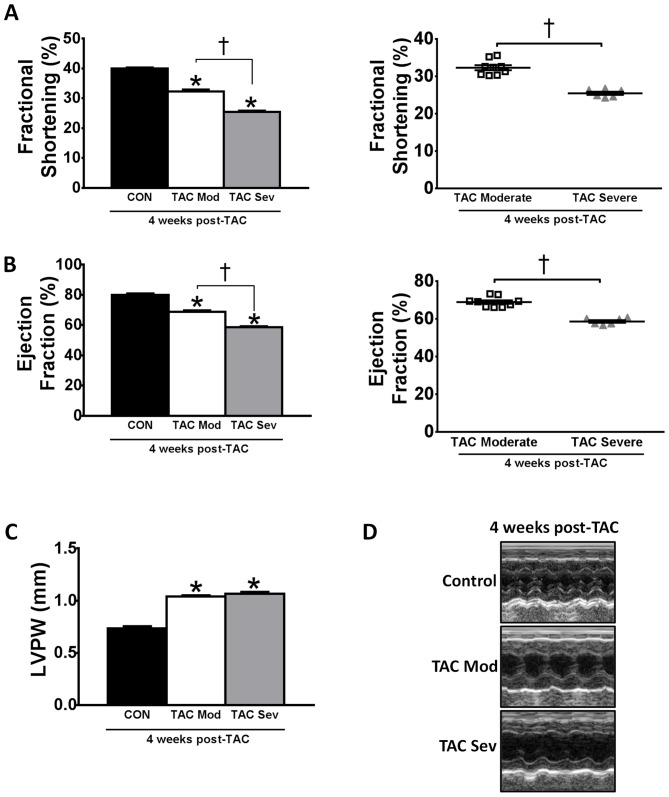
Mice with moderate or severe cardiac dysfunction following four weeks of pressure overload. Quantification of (A) fractional shortening, (B) ejection fraction and (C) left ventricular posterior wall thickness (LVPW) at four weeks post-TAC in control (CON), TAC moderate (TAC mod) and TAC severe (TAC sev) mice. Scatter plots demonstrate no overlap of fractional shortening and ejection fraction between TAC moderate and TAC severe groups. (D) Representative M-mode echocardiograms. N = 4–9 per group. *P<0.05 vs. control at four weeks post-TAC. †P<0.05. 1 way ANVOA followed by Fisher’s Post Hoc Test.

To determine whether inhibition of miR-34a could improve systolic function in a mouse model with pre-existing pathological hypertrophy and systolic dysfunction, we subjected mice to TAC for four weeks. After four weeks of pressure overload, LV remodeling was confirmed by echocardiography. On the basis of the echocardiography results measured four weeks after TAC (and before administration of LNA-control or LNA-antimiR-34a), two distinct groups of mice became apparent: 1) mice with a moderate hypertrophic cardiomyopathy phenotype, i.e. increased wall thickness, and moderate cardiac dysfunction (fractional shortening >30% but significantly less than control unoperated mice, Figure 1, Table 1); and 2) mice with a more severe cardiac hypertrophic cardiomyopathy; i.e. increased wall thickness and more significant cardiac dysfunction (fractional shortening <30%, Figure 1, Table 1). Thus, mice were classified as having a moderate or severe cardiomyopathy phenotype based on echocardiography parameters prior to treatment (subsequently referred to as TAC moderate and TAC severe, respectively). The reason the TAC surgery resulted in mice with either a moderate or severe pathology is not apparently obvious but is consistent with our previous report [31].

**Table 1:** Echocardiography data of control and TAC mice at baseline and four weeks post-TAC.

	Baseline			Four weeks post-TAC		
	Control	TAC moderate	TAC severe	Control	TAC moderate	TAC severe
**Number of animals**	4	9	6	4	9	6
**BW (g)**	28.6±0.4	28.1±0.8	27.4±1.1	30.3±0.7	29.2±0.5	29.0±0.6
**Heart rate, bpm**	608±9	567±13	555±28	643±21	597±15	580±17
**LVPW, mm**	0.67±0.01	0. 69±0.01	0.73±0.03	0.73±0.02	1.04±0.01*	1.06±0.02*
**IVS, mm**	0.75±0.01	0.74±0.01	0.73±0.01	0.78±0.01	1.15±0.02*	1.19±0.02*†
**LVEDD, mm**	3.81±0.12	4.01±0.08	4.01±0.11	3.89±0.21	4.03±0.10	4.34±0.13
**LVESD, mm**	2.23±0.08	2.40±0.07	2.41±0.08	2.33±0.11	2.73±0.08*	3.24±0.11*†
**FS, %**	41±1	40±1	40±1	40±0	32±1*	25±0*†
**EF, %**	80±1	79±1	78±1	78±0	69±1*	59±1*†

BW, body weight; LV, left ventricular; LVPW, LV posterior wall thickness; IVS, interventricular septum thickness; LVEDD, LV end-diastolic dimension; LVESD, LV end-systolic dimension; FS, fractional shortening; EF, ejection fraction. Data are shown as mean ± SEM. One way ANOVA followed by Fisher’s Post hoc Test. *P<0.05 vs. baseline of same group and control at same time point, †P<0.05 vs. TAC moderate at same time point.

TAC moderate mice displayed a 20% and 13% decrease in fractional shortening and ejection fraction respectively, compared to the performances of hearts evaluated pre-surgery (Figure 1A & B, Table 1). In contrast, the hearts of TAC severe mice displayed a greater decrease in fractional shortening (37%) and ejection fraction (25%) (Figure 1A & B, Table 1). There was no overlap in fractional shortening or ejection fraction from individual mice in the two groups (Figure 1A & B). Mice with moderate and severe cardiomyopathy were further subdivided and randomized into LNA-control or LNA-antimiR-34a treatment groups. Moderate and severe aortic-banded mice received three consecutive daily injections (subcutaneously) of LNA-oligonucleotides and were then left for eight weeks (Figure S1).

### Inhibition of miR-34a had modest protective actions in a moderate but not severe model of pressure overload

We previously showed that three consecutive daily subcutaneous injections of an LNA-antimiR in mice is sufficient to silence miRNA expression in the heart for at least two months [10]. At eight weeks post-treatment, we confirmed that miR-34a was effectively silenced in heart tissue of TAC moderate and TAC severe LNA-antimiR-34a mice compared to LNA-control mice (Figure S2).

**Figure 2:**
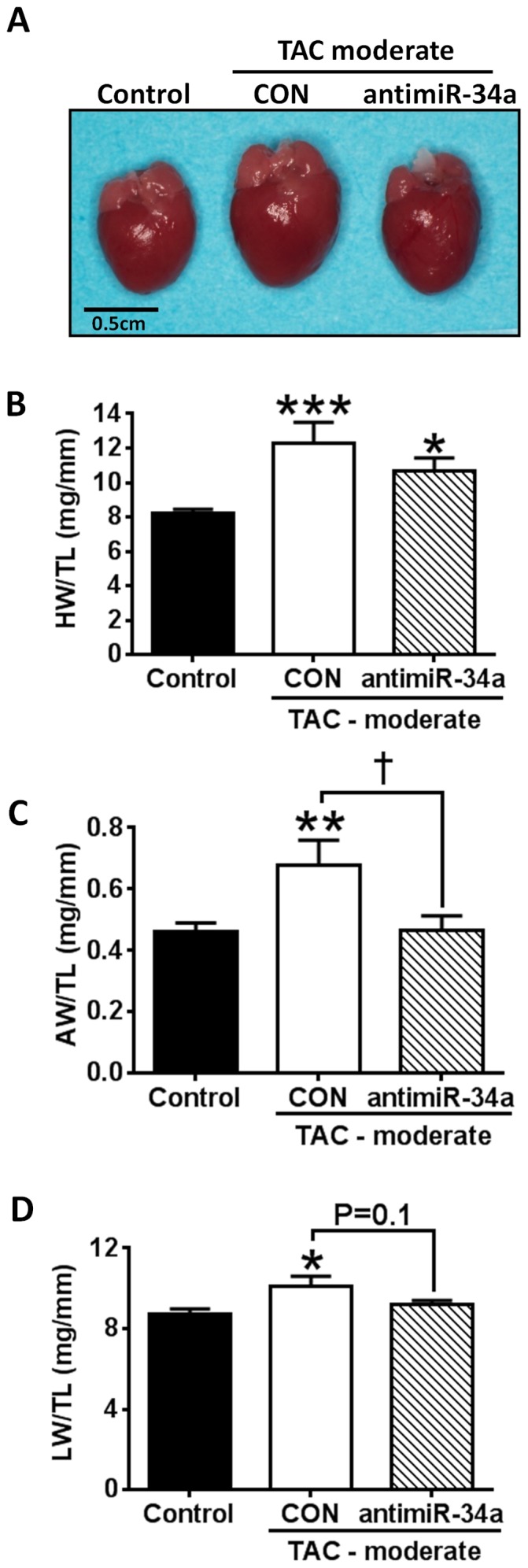
LNA-antimiR-34a is associated with modest improvements in cardiac remodeling in a moderate model of pressure overload. (A) Representative heart images of control, TAC moderate LNA control (CON) and TAC moderate LNA-antimiR-34a (anti-34a) at dissection. Scale bar  =  0.5 cm. (B-D) Graphs of HW/TL, AW/TL and LW/TL. N = 4–8 per group. ***P<0.001 vs. control, **P<0.01 vs. control, *P<0.05 vs. control, †P<0.05. 1 way ANVOA followed by Fisher’s Post Hoc Test.

Compared to control mice, HW/TL increased more significantly in LNA-control TAC moderate mice (∼50%) than LNA-antimiR-34a TAC moderate mice (∼30%; Figure 2B, Table 2). Furthermore, while TAC moderate LNA-control mice displayed significant atrial enlargement (47% increase in atrial weight/TL, AW/TL, Figure 2C, Table 2) and developed lung congestion (15% increase in lung weight/TL, LW/TL, Figure 2D, Table 2), there was no significant increase in AW/TL and LW/TL with LNA-antimiR-34a treatment in TAC moderate mice (Figure 2C & D, Table 2). Therefore, LNA-antimiR-34a treatment attenuated, in part, adverse cardiac remodeling in a setting of moderate pathology.

**Table 2:** Morphological data for control and TAC moderate and severe mice following four weeks of pressure overload and eight weeks after treatment with LNA-control or LNA-antimiR-34a.

	Control	TAC moderate		TAC severe	
		LNA control	LNA antimiR-34a	LNA control	LNA antimiR-34a
**Number of animals**	8	5	4	3	3
**BW (g)**	33.8±0.7	32.3±1.0	31.1±1.0	33.5±1.2	28.3±2.7*§
**TL (mm)**	16.2±0.1	16.2±0.0	16.1±0.1	16.5±0.1	16.1±0.2
**HW (mg)**	133.6±3.7	198.8±20.0‡	172.1±11.1*	285.8±5.6‡∥	307.0±14.1‡∥
**AW (mg)**	7.5±0.5	11.0±1.3#	7.5±0.7**	26.5±1.8†∥	47.3±13.6‡§∥
**LW (mg)**	141.9±4.1	163.2±8.4#	148.2±3.9	398.8±34.2‡∥	393.4±59.0‡∥
**HW/TL (mg/mm)**	8.25±0.22	12.28±1.23‡	10.70±0.73*	17.34±0.32‡∥	19.07±1.00‡∥
**AW/TL (mg/mm)**	0.46±0.03	0.68±0.08#	0.47±0.05**	1.61±0.11†∥	2.93±0.84‡§∥
**LW/TL (mg/mm)**	8.76±0.23	10.09±0.52#	9.20±0.22	24.17±1.90‡∥	24.50±4.00‡∥

BW: body weight, HW: heart weight, AW: atria weight, LW: lung weight, TL: tibia length, HW/TL: heart weight/ tibia length ratio, AW/TL: atria weight/ tibia length ratio, LW/TL: lung weight/ tibia length ratio. Data are shown as mean ± SEM. One-way ANOVA followed by Fisher’s post-hoc test (comparing 5 groups): *P<0.05 vs. control, †P<0.05 vs. control, ‡P<0.001 vs. control, §P<0.05 vs. TAC LNA control of the same group, ∥P<0.05 vs. TAC-moderate of the same treatment group. One-way ANOVA followed by Fisher’s post-hoc test (comparing TAC moderate groups to control, i.e. 3 groups only): #P<0.05 vs. control, **P<0.05 vs. TAC LNA control.

**Figure 3:**
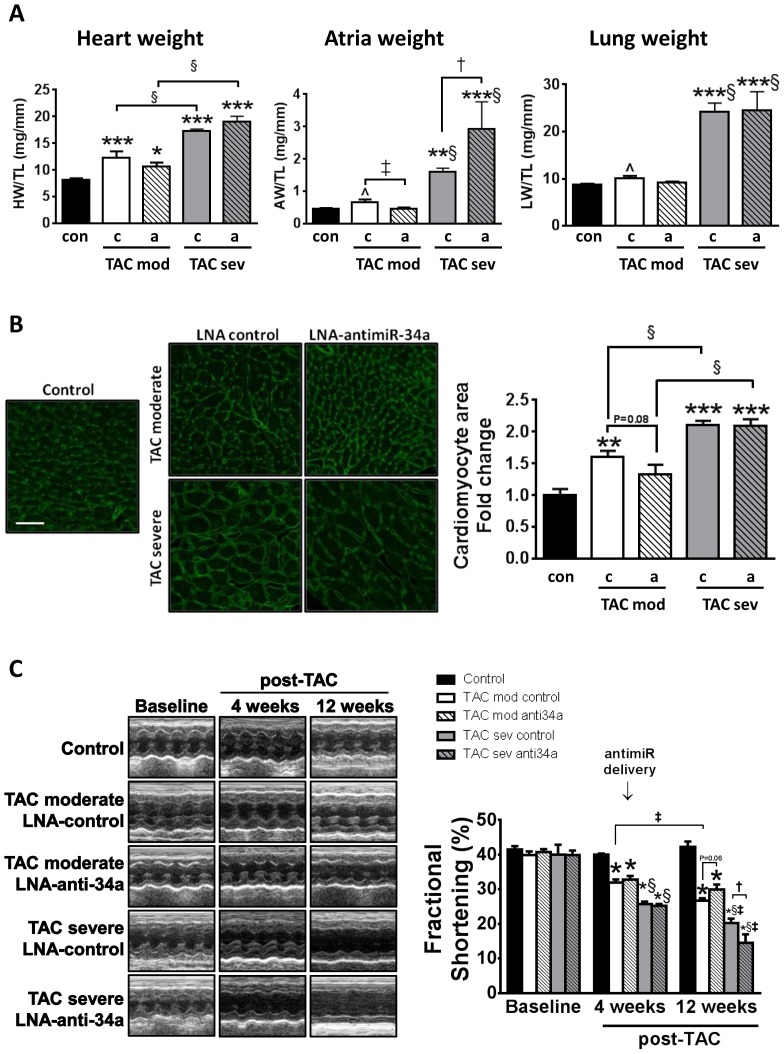
LNA-antimiR-34a maintains cardiac function in a moderate model of pressure overload, but not in a severe model. (A) Graphs of HW/TL, AW/TL and LW/TL in control, TAC moderate (TAC mod) and TAC severe (TAC sev) LNA-control (c) and LNA-antimiR-34a (a) groups. N = 3–8 per group. ***P<0.001 vs. control, **P<0.01 vs. control, *P<0.05 vs. control, †P<0.05, §P<0.05 vs. TAC-moderate of same treatment group. 1 way ANOVA followed by Fisher’s Post Hoc Test (comparing all five groups). When performing 1 way ANOVA followed by Fisher’s Post Hoc Test on control and TAC moderate groups only (i.e. comparing three groups), n = 4–8 per group, ∧P<0.05 vs. control, ‡P<0.05. (B) Representative LV cross-sections stained with wheat germ agglutinin from control and TAC moderate and severe LNA-control and LNA-antimiR-34a mice, and quantification of cell area. Scale bar  =  50 µM. Data are expressed as mean ± SEM. N  =  3–5 per group. **P<0.01 vs. control, ***P<0.001 vs. control, §P<0.05. 1 way ANOVA followed by Fisher’s Post Hoc Test (comparing all five groups). (C) Quantification of fractional shortening at baseline (pre-surgery), four weeks post TAC (before LNA oligonucleotide administration) and 12 weeks post (i.e. eight weeks post treatment) and representative M-mode echocardiograms. N = 4–5 per group. *P<0.05 vs. baseline of the same groups and control at the same time point, †P<0.05, ‡P<0.05 vs. same group at 4 weeks post TAC, §P<0.05 vs. TAC-moderate at same time point. 1 way ANOVA followed by Fisher’s Post Hoc Test.

By contrast, LNA-antimiR-34a treatment provided no protection in those mice classified as having a severe hypertrophic cardiomyopathy phenotype four weeks post TAC. Severe TAC mice administered LNA-control or LNA-antimiR-34a displayed significant increases in HW/TL (110–130% increase vs. control, Figure 3A, Table 2), developed significant atrial enlargement (Figure 3A, Table 2) and lung congestion (increased LW/TL, Figure 3A, Table 2); each significantly greater than that observed in TAC moderate mice (Figure 3A). In the TAC severe model, treatment with LNA-antimiR-34a was unable to attenuate hypertrophy, atrial enlargement and lung congestion, demonstrating that LNA-antimiR-34a was not protective in a severe disease setting (Figure 3A, Table 2). The changes in heart size in both moderate and severe TAC groups were accompanied by similar changes in cardiomyocyte size (Figure 3A and B).

After four weeks of pressure overload, fractional shortening decreased by ∼20% in TAC moderate mice and by ∼37% in TAC severe mice (Figure 3C, Table 3). At eight weeks post-treatment (i.e. 12 weeks post-TAC), fractional shortening decreased further in the TAC moderate LNA-control mice compared with pretreatment values at four weeks post-TAC (Figure 3C, Table 3). However, treatment with LNA-antimiR-34a prevented further deterioration as reflected in the fractional shortening after 12 weeks of TAC compared to pretreatment values at four weeks post-TAC. Furthermore, fractional shortening tended to be higher (P = 0.06) in the moderate TAC antimiR-34a treated mice at 12 weeks post-TAC compared with LNA-control mice at the same time point (Figure 3C, Table 3). In contrast, fractional shortening in the severe model of pressure overload was further decreased at eight weeks post-treatment (i.e. 12 weeks post-TAC) in both TAC LNA-control and TAC LNA-antimiR-34a mice compared with pretreatment values at four weeks post-TAC (Figure 3C, Table 3). Interestingly, fractional shortening in TAC severe LNA-antimiR-34a treated mice was significantly decreased at 12 weeks post-TAC compared to TAC severe LNA-control mice at the same time point (Figure 3C, Table 3). Finally, fractional shortening was further compromised in TAC severe groups compared to TAC moderate groups at 12 weeks post-TAC (Figure 3C, Table 3). Thus, treatment with antimiR-34a prevented further deterioration in cardiac function in a moderate model of pressure overload, but was unable to improve or maintain cardiac function in a severe model of pressure overload.

**Table 3:** Echocardiography data of control and TAC mice at baseline, four weeks post-TAC and eight weeks after treatment with either LNA-control or LNA-antimiR-34a.

	Baseline					Four weeks post-TAC					Eight weeks post treatment				
	Control	TAC Moderate		TAC severe		Control	TAC Moderate		TAC Severe		Control	TAC Moderate		TAC Severe	
		LNA control	LNA antimiR-34a	LNA control	LNA antimiR-34a		LNA control	LNA antimiR-34a	LNA control	LNA antimiR-34a		LNA control	LNA antimiR-34a	LNA control	LNA antimiR-34a
Number of animals	4	5	4	3	3	4	5	4	3	3	4	5	4	3	3
BW (g)	28.6±0.4	28.5±1.3	27.5±0.7	27.3±2.1	27.6±1.0	30.3±0.7	29.6±0.7	28.7±0.6	29.7±0.8	28.4±0.7	34.7±0.9	32.3±0.8	30.7±1.1	33.9±0.9	28.1±2.3†
Heart rate, bpm	608±9	564±22	570±15	500±29	611±11	643±21	584±19	613±25	572±31	587±20	598±20	569±18	597± 11	573±11	614±49
LVPW, mm	0.67±0.01	0.71±0.01	0.66±0.01	0.70±0.05	0.75±0.02	0.73±0.02	1.05±0.00*	1.03±0.02*	1.05±0.02*	1.08±0.04*	0.78±0.02*	1.17±0.03*‡	1.07±0.02*†	1.21±0.02*#§	1.22±0.01*‡§
IVS, mm	0.75±0.01	0.74±0.01	0.74±0.01	0.73±0.01	0.73±0.01	0.78±0.01	1.17±0.02*	1.12±0.01*	1.22±0.03*	1.17±0.04*	0.81±0.02	1.18±0.04*	1.09±0.02*†	1.19±0.03*	1.25±0.02*‡§
LVEDD, mm	3.81±0.12	4.11±0.09	3.89±0.12	4.20±0.11	3.82±0.11	3.89±0.21	4.16±0.15	3.87±0.07	4.45±0.21	4.23±0.18	3.75±0.17	4.33±0.11	4.12±0.14	4.80±0.30*§	5.31±0.08*†‡§
LVESD, mm	2.23±0.08	2.47±0.10	2.39±0.13	2.50±0.10	2.30±0.11	2.33±0.11	2.83±0.12*	2.60±0.07∥	3.31±0.19*§	3.16±0.14*§	2.18±0.14	3.18±0.10*‡	2.89±0.15*#(P = 0.08)	3.82±0.18*#§	4.54±0.11*†‡§
FS, %	41±1	40±1	41±1	40±3	40±1	40±0	32±1*	33±1*	26±1*§	25±1*§	42±2	27±1*‡	30±1* #(P = 0.06)	20±1*#§	14±2*†‡§
EF, %	80±1	78±1	79±1	78±3	78±1	78±0	69±1*	68±1*	59±1*§	58±1*§	81±2	61±1*‡	65±2*†	49±2*#§	37±5*†‡§

BW, body weight; LV, left ventricular; LVPW, LV posterior wall thickness; IVS, interventricular septum thickness; LVEDD, LV end-diastolic dimension; LVESD, LV end-systolic dimension; FS, fractional shortening.

Data are shown as mean ± SEM. One way ANOVA followed by Fisher’s Post hoc Test.

*P<0.05 vs. baseline of same group and control at same time point, †P<0.05 vs. TAC LNA control of same group at same time point, ‡P<0.05 vs. same group at 4 weeks post TAC,§P<0.05 vs. TAC moderate of same treatment group at same timepoint, ∥P<0.1 vs. baseline of same group and control at same time point, #P<0.1 vs. TAC LNA control of same group at same time point.

### Inhibition of miR-34a did not alter the expression of cardiac stress genes following pressure overload

**Figure 4:**
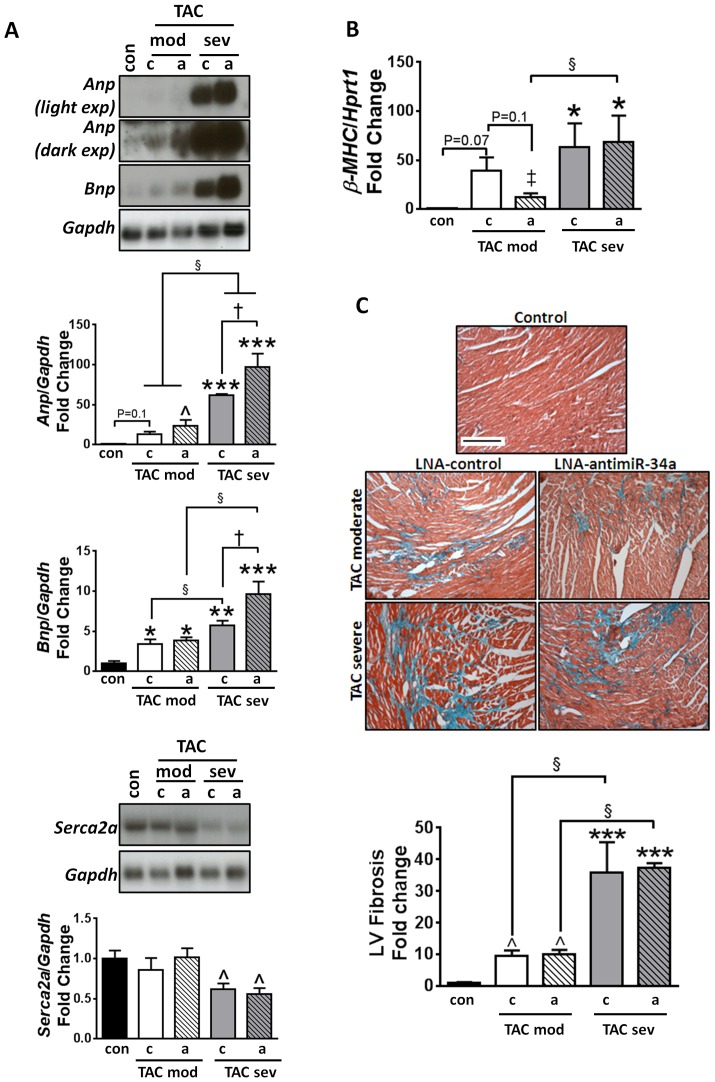
Cardiac stress gene expression and fibrosis in moderate and severe models of pressure overload. (A) Northern blots and quantification of *Anp*, *Bnp*, and *Serca2a* relative to *Gapdh*, in hearts of control (con), TAC moderate (mod), TAC severe (sev) mice dosed with either LNA-control (c) or LNA-antimiR-34a (a). For Northern blot of *Anp*, a light exposure (light exp) and dark exposure (dark exp) has been included. N = 3–5 per group. ***P<0.001 vs. control, **P<0.01 vs. control, *P<0.05 vs. control, §P<0.05, †P<0.05, 1 way ANOVA followed by Fisher’s Post Hoc Test (comparing all five groups). When performing 1 way ANOVA followed by Fisher’s Post Hoc Test on control and TAC severe groups only (comparing three groups), N = 3 per group, or control and TAC moderate groups only (comparing three groups), N = 3–5 per group, ∧P<0.05 vs. control. (B) qPCR of *β-MHC* relative to *Hprt1* in control, TAC moderate and TAC severe mice dosed with LNA-control or LNA-antimiR-34a. N = 3–5 per group. *P<0.05 vs. control, §P<0.05, ‡P<0.05 vs. control, 1 way ANOVA followed by Fisher’s Post Hoc Test (comparing all 5 groups). (C) LV cross-sections stained with Masson’s trichrome and quantification of LV fibrosis in control, TAC moderate and TAC severe mice dosed with LNA-control or LNA-antimiR-34a. Scale  =  200 µM. N = 4–5 per group. ***P<0.001 vs. control, §P<0.05 vs. TAC-moderate of the same treatment group, 1 way ANOVA followed by Fisher’s Post Hoc Test (comparing all five groups). When comparing control and TAC moderate groups only (comparing three groups), N = 4–5 per group, ∧P<0.05 vs. control, 1 way ANOVA followed by Fisher’s Post Hoc Test.

Pathological hypertrophy is typically associated with the re-expression of the cardiac fetal gene program, accumulation of collagen in the extracellular matrix (interstitial fibrosis) and down-regulation of calcium handling proteins [32]. Cardiac dysfunction in TAC moderate and TAC severe LNA-control treated mice was accompanied by increased expression of fetal genes, including atrial natriuretic peptide (*Anp*), B type natriuretic peptide (*Bnp*) and β myosin heavy chain (*β-MHC*), which were exacerbated in the TAC severe model (Figure 4A). Although *Anp* and *Bnp* were not attenuated with LNA-antimiR-34a treatment in moderate and severe TAC groups, there was a trend for decreased *β-MHC* in TAC moderate mice treated with LNA-antimiR-34a, but not in TAC severe mice treated with LNA-antimiR-34a (Figure 4B).

There was no change in sarcoplasmic/endoplasmic reticulum Ca^2+^-ATPase 2a (*Serca2a*) gene expression in TAC moderate LNA-control or LNA-antimiR-34a treated mice, most likely as severe cardiac dysfunction was not present (Figure 4A). In contrast, TAC severe LNA-control and LNA-antimiR-34a mice exhibited significantly decreased *Serca2a* expression, which was not different between treatment cohorts (Figure 4A). Decreased *Serca2a* expression is a hallmark of heart failure that results in abnormal calcium handling. The disturbances in calcium metabolism contribute significantly to the contractile dysfunction observed in heart failure [33], [34], which may explain why mice exhibiting altered *Serca2a* expression have more severe cardiac dysfunction.

Pathological hypertrophy is associated with progressive accumulation of collagen in the interstitial space which adversely influences contractile performance. There was a significant increase in LV fibrosis in TAC moderate mice, independent of treatment, which was further increased in TAC severe mice (Figure 4C). Treatment with LNA-antimiR-34a did not attenuate fibrosis in either the TAC moderate or TAC severe groups (Figure 4C).

### Impact of miR-34a inhibition on the expression of validated targets

**Figure 5:**
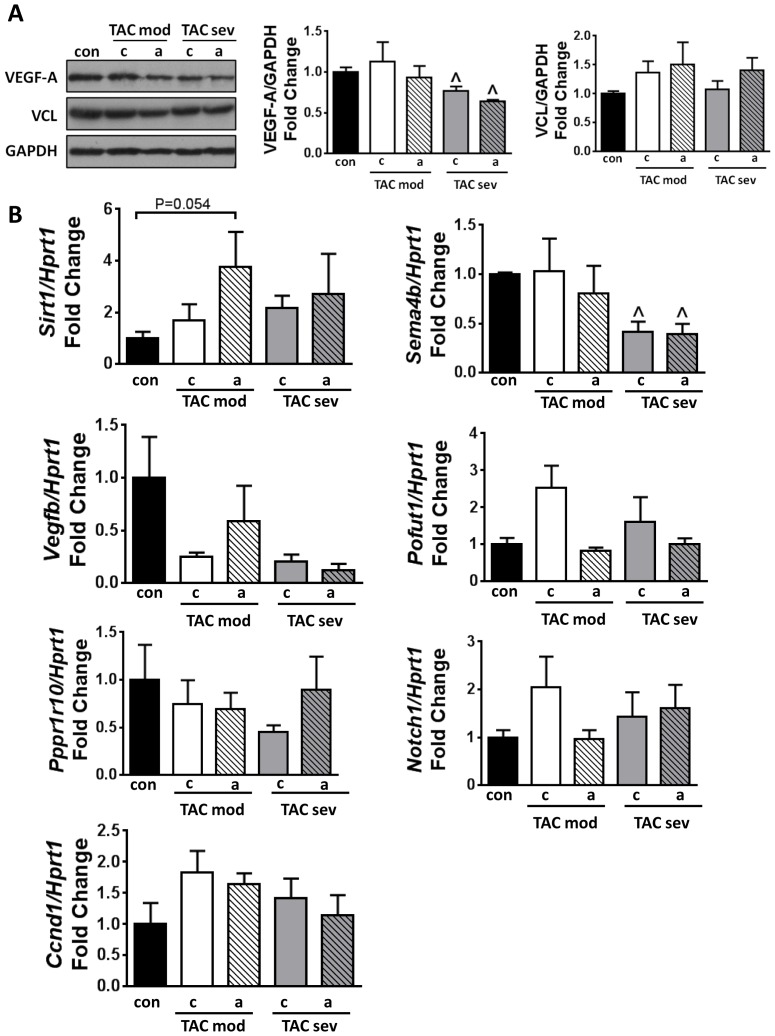
Analysis of miR-34a target gene or protein expression. (A) Representative Western blots and quantification of VEGF-A and VCL relative to GAPDH in hearts of control (con), TAC moderate (mod), TAC severe (sev) mice dosed with either LNA-control (c) or LNA-antimiR-34a (a). N = 3–5 per group. ∧P<0.05 vs. control when using 1 way ANOVA followed by Fisher’s Post Hoc test on severe group only. (B) qPCR analysis of *Sirt1, Sema4b*, *Vegfb, Pofut1,* PNUTS *(Pppr10)*, *Notch 1* and Cyclin D1 *(Ccnd1)* relative to *Hprt1*. N = 3–5 per group. ∧P<0.05 vs. control when using 1 way ANOVA followed by Fisher’s Post Hoc test on control and TAC severe groups only (comparing three groups).

Identified targets of miR-34a and the miR-34 family which have been associated with improved cardiac outcomes due to their roles related to cell survival, proliferation, cardiac repair and regeneration, maintenance of cardiac function, and angiogenesis include vinculin (*Vcl*), phosphatase 1 nuclear targeting subunit (also known as PNUTS), vascular endothelial growth factor–A and B (*Vegfa, Vegfb*), Cyclin D1, Sirt1, Notch1, protein O-fucosyltransferase 1 (*Pofut1*) and semaphorin 4b (*Sema4b*) [10], [11]. In this study, there was a trent for increased *Sirt1* mRNA levels in TAC moderate LNA-antimiR-34a hearts when compared to control hearts (Figure 5B). However, protein levels of vinculin (VCL), and mRNA levels of PNUTS *(Pppr1r10)*, *Notch1, Pofut1,* Cyclin DI *(Ccnd1), Vegfb* and *Sema4b*, did not change in either the TAC moderate or TAC severe models of pressure overload with LNA-antimiR-34a treatment (Figure 5A & B). Protein levels of VEGF-A, which were associated with increased capillary density in our previous report (compared to TAC LNA-control mice) [10], did not increase with LNA-antimiR-34a treatment in TAC moderate or TAC severe mice. The lower levels of VEGF-A protein and *Sema4b* mRNA in hearts of TAC severe mice compared with control mice are consistent with the more severe cardiomyopathy phenotype in these animals (Figure 5A). In summary, treatment with LNA-atimiR-34a was unable to attenuate the expression of cardiac stress and fibrotic genes, improve/maintain *Serca2a* expression and significantly derepress validated targets in both moderate and severe mouse models of pressure overload induced by TAC.

### Expression of miR-34 family members, miR-34b and miR-34c, is elevated in moderate and severe models of pressure overload

**Figure 6:**
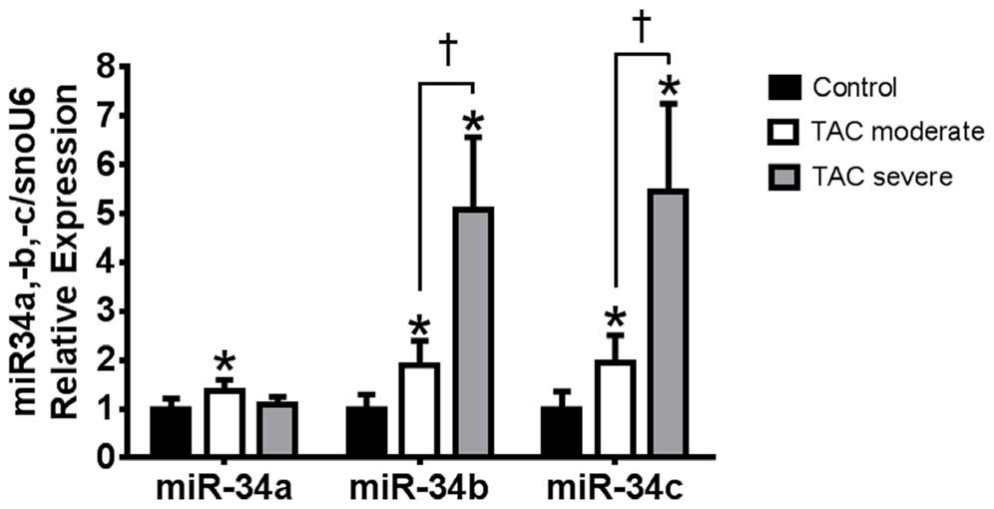
Expression of miR-34a, miR-34b and miR-34c in control and TAC mice. qPCR of miR-34a, miR-b and miR-34c in hearts of control, TAC moderate and TAC severe mice. N = 3–5 per group. *P<0.05 vs. control, †P<0.05. 1 way ANOVA followed by Fisher’s Post Hoc test.

We have previously shown that expression of other miR-34 family members, miR-34b and miR-34c, are elevated in settings of cardiac stress [10], [18]. Since we identified only modest protection in the moderate model of TAC, and no protection in the severe model of TAC with LNA-antimiR-34a administration in the present study, we analyzed the expression of the other miR-34 family members (i.e. miR-34b and -34c) in control mice, TAC moderate LNA-control and TAC severe LNA-control mice. There was a significant increase in 34a, miR-34b and miR-34c in TAC moderate LNA-control mice compared to control mice (Figure 6). Interestingly, only miR-34b and miR-34c were significantly increased in TAC severe LNA-control mice (and at higher levels compared to TAC moderate), but not miR-34a. This may explain the partial protection observed in TAC moderate mice, but no protection in TAC severe mice, given that only miR-34a was inhibited in this study (Figure 6). We have previously shown that LNA-antimiR-34a does not significantly silence miR-34b and miR-34c in the heart [10].

## Discussion

In a previous study, we reported that inhibition of the miR-34 family attenuated LV remodeling and atrial enlargement in mouse models with established cardiac dysfunction due to MI or pressure overload [10]. Interestingly, inhibition of miR-34a alone provided no significant benefit in the MI setting, but was not explored in the pressure overload model [10]. Given that inhibition of miR-34a is being considered as a possible therapy for cardiovascular disease [11], [21], [22], we set out to determine whether miR-34a inhibition could provide benefit in a mouse model of pressure overload-induced pathological hypertrophy. A potential advantage of inhibiting miR-34a alone, as opposed to the entire family, is related to fewer possible off-target effects.

The major finding of the present study is that inhibition of miR-34a provided some protection in the TAC moderate model, but not in the TAC severe model. LNA-antimiR-34a treated TAC moderate mice did not develop lung congestion, atrial enlargement was attenuated, and importantly, there was no further deterioration in cardiac function at 12 weeks post TAC compared to pretreatment values at four weeks post TAC. In contrast, systolic function continued to fall in TAC moderate LNA-control mice. HW/TL increased by approximately 49% in TAC moderate LNA-control mice, and only 30% in TAC moderate LNA-antimiR-34a mice. Despite attenuation of adverse cardiac remodeling, treatment with LNA-antimiR-34a was not associated with a more favorable cardiac molecular signature or less fibrosis. The only parameter which showed a tendency to be attenuated in antimiR-34a treated mice was β-MHC expression. Treatment with LNA-antimiR-34a was associated with a trend for increased *Sirt1* expression. *Sirt1* protects the heart against ageing and stress [35], thus increased *Sirt1* mRNA may contribute to the modest cardiac protection observed in TAC moderate mice. However, treatment with LNA-antimiR-34a was unable to increase the expression of other validated targets that have been associated with improved outcomes in settings of cardiac stress (VEGF-A, VEGF-B, VCL, PNUTS, Cyclin D1). This is in contrast to the more favorable effect of inhibiting the entire miR-34 family in the pressure overload mouse model in our previous report [10]. Inhibition of the entire miR-34 family improved cardiac function, and this was associated with reduced fibrosis, decreased *Anp* expression, increased angiogenesis, maintenance of *Serca2a* expression, and up-regulation of several direct targets, including VEGFs, Pofut1, Notch1 and Sema4b [10]. As miRNAs target several hundred mRNAs, it is possible that other targets could contribute to the modest cardiac benefit in the moderate model of pressure overload with LNA-antimiR-34a treatment. Finally, inhibition of miR-34a was not protective in a severe model of pressure overload since treatment with LNA-antimiR-34a did not attenuate LV remodeling, improve cardiac function or cardiac stress gene expression, and fibrosis. This finding is consistent with our previous report in which inhibition of miR-34a did not prevent LV remodeling or improve cardiac function following chronic MI [10]. Collectively, these studies suggest that inhibition of miR-34a provides some benefit in an acute setting of cardiac stress or conditions with moderate pathology, but not in chronic or severe settings.

A possible explanation for the reduced capacity of LNA-antimiR-34a to provide protection in chronic or severe settings of cardiac pathology may be due to increased expression of two other miR-34-family members, miR-34b and miR-34c (ranging from a ∼1.7 to 4-fold increase in MI or TAC [10]). We have previously shown that LNA-antimiR-34a does not inhibit miR-34b and miR-34c [10], which were both elevated in the TAC severe model in this study. Interestingly, miR-34a was elevated in the hearts of the TAC moderate but not TAC severe model. Thus, this could explain why pharmacologic inhibition was not effective in the TAC severe model, and only partially protective in the TAC moderate model of pressure overload, which had increased levels of all miR-34 family members (i.e. miR-34a, miR-34b and miR-34c).

miRNA-based therapies are advancing at a rapid rate, with successful completion of phase 1 and phase 2 clinical trials of Santaris Pharma’s LNA-modified antimiR, miravirsen, targeting miR-122 for the treatment of hepatitis C virus infection [9]. Thus, there is great enthusiasm for the development of miRNA-based therapies for cardiovascular disease. The different degrees of efficacy upon inhibition of miR-34a in different cardiac disease models highlights the importance of assessing therapeutic targeting of candidate miRNAs over a broad spectrum of cardiac conditions, as some miRNAs may be more effective than others under particular settings (e.g. acute vs. chronic). In recognition that cardiovascular disease and cardiac remodeling is associated with simultaneous dysregulation of several miRNAs (e.g. miR-1, miR-34a, miR-133, miR-199b, miR-320 [11], [15], [36]–[38]) or miRNA families (e.g. miR-34 family [10], miR-208 family [39]), tiny 8-mer seed-targeting LNA-antimiRs could provide an advantage by simultaneous inhibition of entire miRNA seed families [10], [27]. The current study and our previous work [10] highlight a different therapeutic benefit of inhibiting a single miRNA (miR-34a) or a miRNA family (miR-34 family) in moderate and severe models of sustained cardiac stress. Greater therapeutic benefit of inhibiting the entire miR-34 family may be related to the regulation of more target genes. As shown by miRNA target prediction databases based on different algorithms (MiRanda, DIANA, PicTar), the miR-34 family is predicted to repress 24–40% (mouse) or 31–55% (human) more mRNAs than miR-34a alone (Figure S3). Thus, targeting miRNA families may be able to regulate multiple additional biological networks. However, this may have both favorable and unfavorable consequences, and the potential off-target effects must be carefully assessed when developing miRNA therapeutics [10].

Although there are several advantages of developing miRNA-based therapeutics, many miRNAs are ubiquitously expressed and miRNA-based therapeutics are taken up by various organs upon systemic delivery, making clinical intervention complex. Whilst inhibition of miR-34a and the miR-34 family is protective in the hearts of mice [10], [11], the effect of prolonged/chronic inhibition of miR-34a and its family members may not be ideal because of its ability to drive tumorigenesis [40], [41], although a recent study has shown that the miR-34 family is not required for tumor suppression in mice [42]. Conversely, miR-34a replacement therapy as a cancer therapeutic may have adverse affects on the heart or render the heart more susceptible to dysfunction in settings of stress. Following successful pre-clinical work that has demonstrated potent anti-tumor effects by introducing miR-34a mimics in different mouse models of cancer [43], [44], Mirna Therapeutics have developed MRX34 to restore the expression of miR-34a in tumor cells and are currently conducting a Phase 1 study in patients with primary liver cancer or metastatic cancer with liver involvement [45], [46]. In addition to the tumor suppressive role of miR-34a, miR-34 expression is important for long-term maintenance of the brain, healthy aging and modulation of protein homeostasis with age in *Drosophila* [47], and miR-34b and miR-34c are key regulators of skeletogenesis [48]. Therefore, while miR-34 has marked therapeutic potential in conditions of heart stress, it will be critical to assess the impact of miR-34 in brain, bone and other organs during its development as a therapeutic agent, or may require the development of cardiac-specific approaches^27, 32, 45,^ [49].

In summary, administration of LNA-antimiR-34a in a moderate model of pressure overload was able to attenuate atrial enlargement, prevent lung congestion and maintain cardiac function, but was unable to attenuate pathological cardiac remodeling or prevent further cardiac dysfunction in a severe model of pressure overload. Thus, drugs that target the entire miR-34 family are likely to have greater therapeutic benefit in settings of sustained cardiac stress and severe pathology than inhibition of miR-34a alone.

## Supporting Information

Figure S1 Experimental Timeline and dosing regimen of LNA-control/antimiR-34a for mice subjected to pressure overload (TAC).
(TIF)

Figure S2 Administration of LNA-antimiR-34a silences miR-34a in the heart. qPCR showing inhibition of miR-34a in hearts of TAC moderate and TAC severe mice dosed with LNA-antimiR-34a vs. LNA-control. N = 3-5 per group. *P<0.05 vs. LNA-control. Unpaired t-test.
(TIF)

Figure S3 Number of predicted targets of miR-34a versus the miR-34 family. Bar graphs showing the number of predicted targets of miR-34a versus the miR-34 family using three target prediction algorithms in mice and humans (MiRanda 4.0, PicTar, DIANA microT v5.0). All miR-34a targets are also predicted targets of miR-34 family.
(TIF)

Table S1 Morphological data for control adult male mice eight weeks after administration of a LNA-control or LNA-antimiR-34a.
(DOCX)
