# Supplementary figures and images for: Silencing of miR-34a Attenuates Cardiac Dysfunction in a Setting of Moderate, but Not Severe, Hypertrophic Cardiomyopathy
Source: PLoS One. 2014 Feb 27;9(2):e90337. doi: 10.1371/journal.pone.0090337 (PMC3937392; doi:10.1371/journal.pone.0090337)

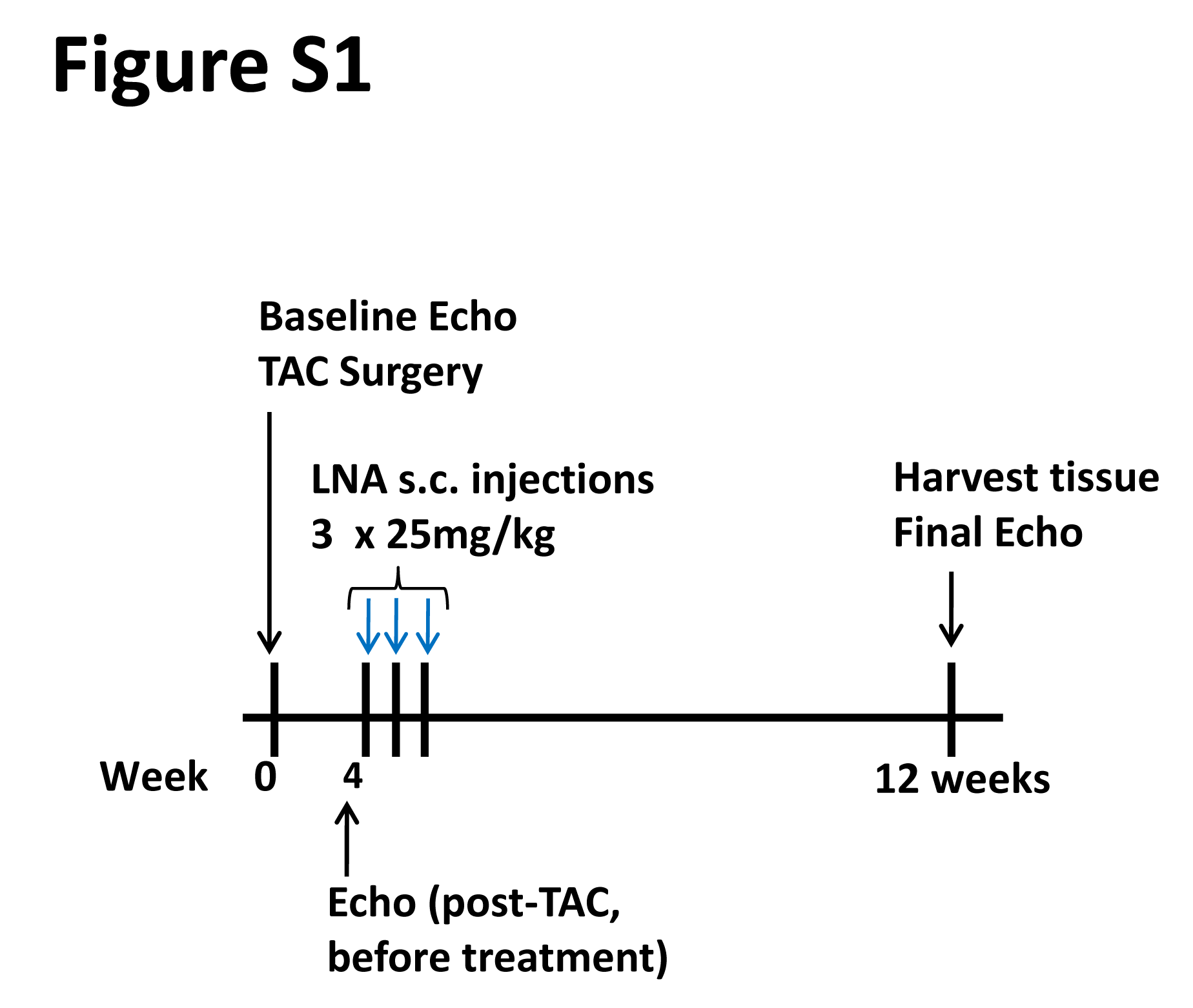

Supplement: Figure S1 — Experimental Timeline and dosing regimen of LNA-control/antimiR-34a for mice subjected to pressure overload (TAC). (TIF) [file pone.0090337.s001.tif]

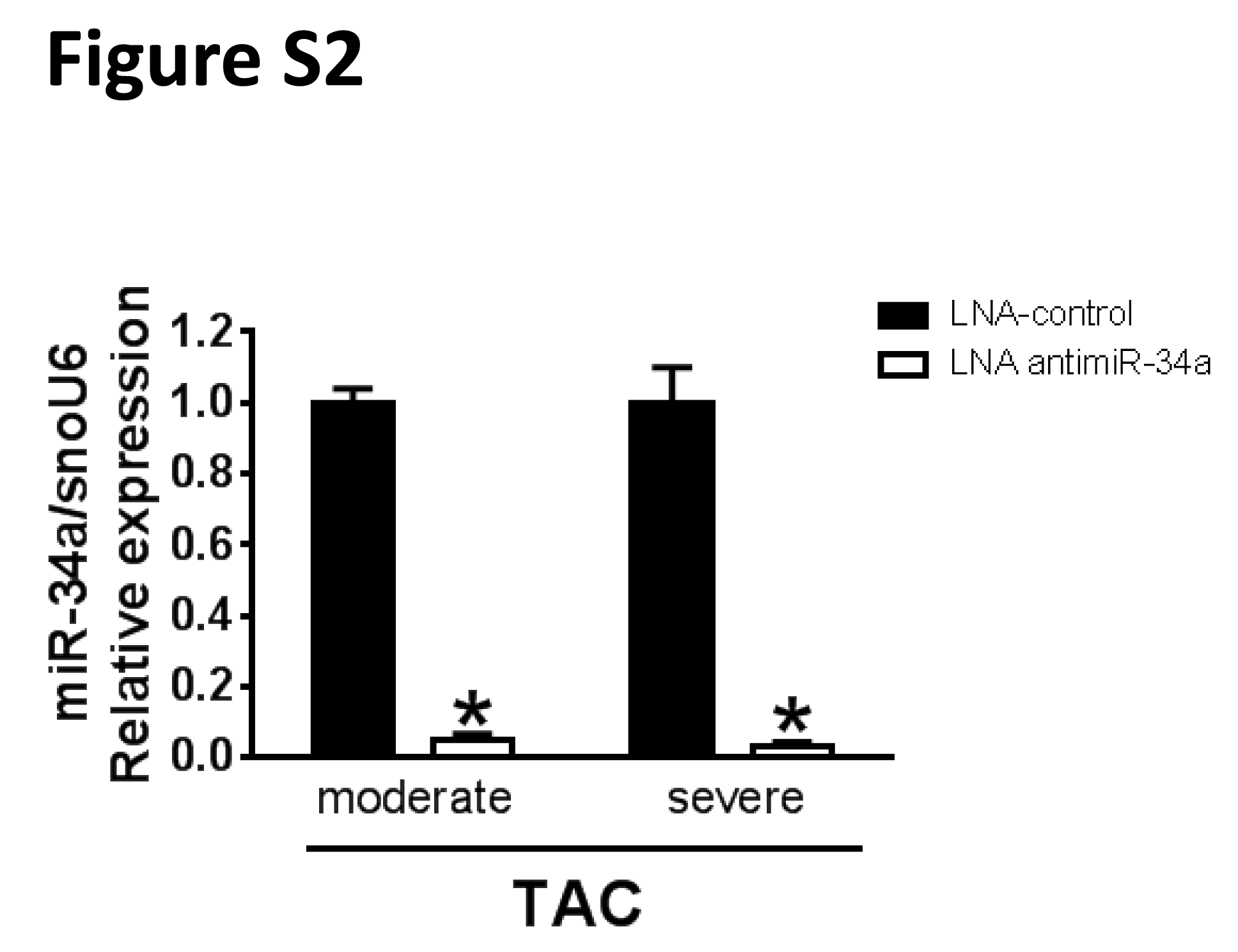

Supplement: Figure S2 — Administration of LNA-antimiR-34a silences miR-34a in the heart. qPCR showing inhibition of miR-34a in hearts of TAC moderate and TAC severe mice dosed with LNA-antimiR-34a vs. LNA-control. N = 3-5 per group. *P<0.05 vs. LNA-control. Unpaired t-test. (TIF) [file pone.0090337.s002.tif]

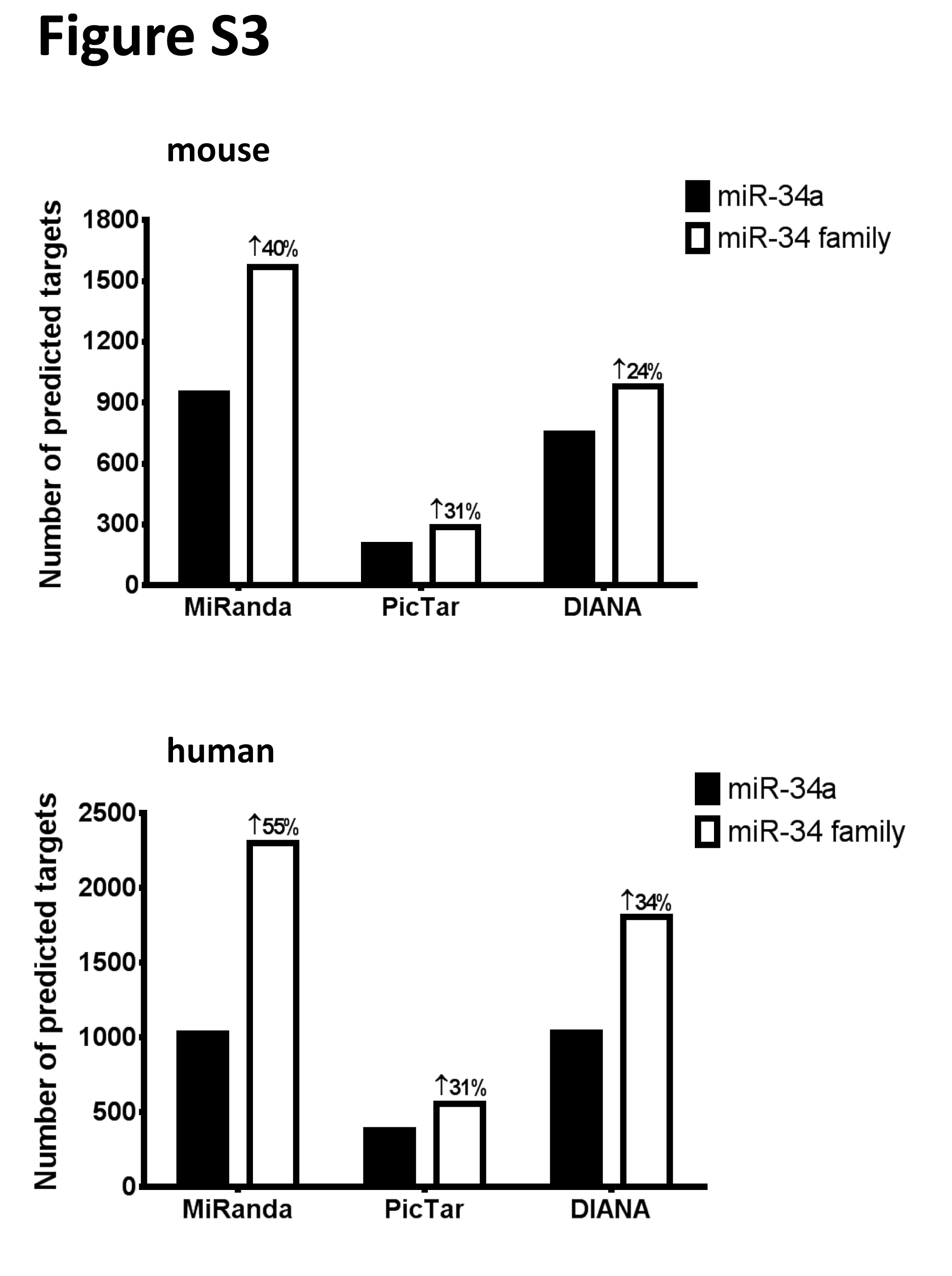

Supplement: Figure S3 — Number of predicted targets of miR-34a versus the miR-34 family. Bar graphs showing the number of predicted targets of miR-34a versus the miR-34 family using three target prediction algorithms in mice and humans (MiRanda 4.0, PicTar, DIANA microT v5.0). All miR-34a targets are also predicted targets of miR-34 family. (TIF) [file pone.0090337.s003.tif]
