# Supplementary material for: Silencing of miR-34a Attenuates Cardiac Dysfunction in a Setting of Moderate, but Not Severe, Hypertrophic Cardiomyopathy
Source: PLoS One. 2014 Feb 27;9(2):e90337. doi: 10.1371/journal.pone.0090337 (PMC3937392; doi:10.1371/journal.pone.0090337)
Supplement: Table S1 — Morphological data for control adult male mice eight weeks after administration of a LNA-control or LNA-antimiR-34a. (DOCX) [file pone.0090337.s004.docx]

Table S1. Morphological data for control adult male mice eight weeks after administration of a LNA-control or LNA-antimiR-34a.

|  | **Control** | |
| --- | --- | --- |
|  | **LNA-control** | **LNA-antimiR-34a** |
| **Number of animals** | 3 | 3 |
| **BW (g)** | 30.0 ± 0.6 | 28.9 ± 1.0 |
| **TL (mm)** | 15.8 ± 0.3 | 15.8 ± 0.2 |
| **HW (mg)** | 126.2 ± 3.9 | 122.3 ± 4.3 |
| **AW (mg)** | 6.1 ± 0.3 | 5.4 ± 0.2 |
| **LW (mg)** | 151.0 ± 5.4 | 143.8 ± 0.9 |
| **HW/TL (mg/mm)** | 7.94 ± 0.11 | 7.73 ± 0.27 |
| **AW/TL (mg/mm)** | 0.39 ± 0.02 | 0.34 ± 0.01 |
| **LW/TL (mg/mm)** | 9.50 ± 0.24 | 9.09 ± 0.17 |

BW: body weight, HW: heart weight, AW: atria weight, LW: lung weight, TL: tibia length, HW/TL: heart weight/ tibia length ratio, AW/TL: atria weight/ tibia length ratio, LW/TL: lung weight/ tibia length ratio. Data are shown as mean ± SEM. Unpaired t-test (no significant difference between groups).
